# Intravitreal Injection of Liposomes Loaded with a Histone Deacetylase Inhibitor Promotes Retinal Ganglion Cell Survival in a Mouse Model of Optic Nerve Crush

**DOI:** 10.3390/ijms21239297

**Published:** 2020-12-06

**Authors:** Mi Sun Sung, Myeong Ju Moon, Reju George Thomas, So Young Kim, Jun Sung Lee, Yong Yeon Jeong, In-Kyu Park, Sang Woo Park

**Affiliations:** 1 Department of Ophthalmology, Chonnam National University Medical School and Hospital, Gwangju 61469, Korea; sms84831@hanmail.net (M.S.S.); syou8246@naver.com (S.Y.K.); 2 Department of Radiology, Biomolecular Theranostics (BiT) Lab, Chonnam National University Medical School, Gwangju 61469, Korea; mjmoon2398@gmail.com (M.J.M.); regeth@gmail.com (R.G.T.); yjeong@jnu.ac.kr (Y.Y.J.); 3 Parangsae Eye Clinic, Gwangju 61469, Korea; letmedream2@gmail.com; 4 Department of Biomedical Sciences, Chonnam National University Medical School, Gwangju 61469, Korea; pik96@jnu.ac.kr

**Keywords:** liposome, histone deacetylase inhibitor, intravitreal injection, retinal ganglion cell, optic nerve crush

## Abstract

Various neuroprotective agents have been studied for the treatment of retinal ganglion cell (RGC) diseases, but issues concerning the side effects of systemically administered drugs and the short retention time of intravitreally injected drugs limit their clinical applications. The current study aimed to evaluate the neuroprotective effects of intravitreally injected trichostatin A (TSA)-loaded liposomes in a mouse model of optic nerve crush (ONC) and determine whether TSA-loaded liposomes have therapeutic potential in RGC diseases. The histone deacetylase inhibitor, TSA, was incorporated into polyethylene glycolylated liposomes. C57BL/6J mice were treated with an intravitreal injection of TSA-loaded liposomes and liposomes loaded with a lipophilic fluorescent dye for tracking, immediately after ONC injury. The expression of macroglial and microglial cell markers (glial fibrillary acidic protein and ionized calcium binding adaptor molecule-1), RGC survival, and apoptosis were assessed. We found that the liposomes reached the inner retina. Their fluorescence was detected for up to 10 days after the intravitreal injection, with peak intensity at 3 days postinjection. Intravitreally administered TSA-loaded liposomes significantly decreased reactive gliosis and RGC apoptosis and increased RGC survival in a mouse model of ONC. Our results suggest that TSA-loaded liposomes may help in the treatment of various RGC diseases.

## 1. Introduction

The first steps of visual processing take place in the retina and the retina contains 6 neural groups. The rod and cone photoreceptors are the light-sensitive neurons, and the bipolar cells, horizontal cells, and amacrine cells are the interneurons. At the innermost layer of the retina, retinal ganglion cells (RGCs) reside, and they are the sole output neurons. All RGCs project their axons toward the optic nerve head, and then this bundle of axonal fibers reaches a number of structures in the brain for transmitting visual information. Because the RGCs bear the sole responsibility of propagating visual stimuli to the brain, their death causes significant visual loss [1]. RGC death is the central and irreversible endpoint of optic neuropathies, including glaucoma, hereditary optic neuropathies, and ischemic optic neuropathies [2,3,4].

Glial cells respond relatively quickly to retinal stress or injury. Microglia become “amoeboid” and exhibit proliferative potential, while macroglial cells such as Müller cells and astrocytes, become hypertrophic rather than undergoing proliferation [5,6]. According to the previous report [5], glial fibrillary acidic protein (GFAP) expression peaked at 7 days after optic nerve crush (ONC), and the expression levels declined at the latter time point. Similarly, the number of microglia increased at 7–14 days after ONC. The ONC-injury-induced gliosis promotes the progressive death of RGCs, which starts as early as 7 days and increases with time. Li et al. [7] reported that maximum RGC loss was detected 3 weeks after ONC.

In recent years, there has been considerable progress in neuroprotective therapeutic strategies to overcome various RGC-related optic neuropathies, and they have shown translational potential in human RGCs [8,9]. However, despite thousands of research studies, attempts to translate the experimental results from the laboratory to the clinic remain in their infancy and their clinical application is limited. One of the barriers is biopharmaceutical problems related to the special characteristics of the eye that restrict drug bioavailability [10,11].

Topical administration of drugs using eyedrops results in their excretion through tear drainage and peribulbar or choroidal blood flow, and as a result, less than 5% of the drug can cross the corneal barrier and gain access to the posterior segment of the eye [11]. Systemically administered drugs also have very low efficiency because of the blood-aqueous barrier and inner and outer blood-retinal barriers, and these drugs are associated with side effects [12]. The best method to deliver drugs to the posterior segment of the eye is by injecting drugs into the vitreous. Intravitreal injections are used widely in clinical practice. However, most of the small molecules injected into the vitreous cavity are eliminated rapidly via aqueous humor or systemic blood circulation, exiting the eye with very short half-lives [13]. Therefore, frequent injections are needed to treat diseases, which is burdensome for both patients and clinicians. Further, there are concerns about the increased risk of cataract development, vitreous hemorrhage, retinal detachment, and endophthalmitis.

In the last decade, several studies have attempted to increase drug retention time in the vitreous cavity and optimize drug delivery for posterior segment eye diseases. Many nanoparticulate-based drug delivery systems, such as nanoparticles, nanoemulsions, nanosuspensions, liposomes, dendrimers, and niosomes have been investigated [14,15,16]. Among these, liposomes have been studied extensively in ophthalmology. Liposomes are small lipid vesicles composed of a phospholipid bilayer, and both water-soluble and lipid-soluble drugs can be incorporated in them. Because of their biocompatibility, easily tunable characteristics, and drug loading capacity, liposomes have been used widely for intravitreal injections of drugs [17,18,19,20,21,22]. Lee et al. [17] reported an optimal liposomal formulation that can diffuse through the vitreous humor, deliver the incorporated agents to retinal layers, and maintain them in the layers for a long time.

Recently, our study group demonstrated the role of histone deacetylase 2 (HDAC2) on glial cell activation and RGC survival in mouse retinas [23]. We found that the inhibition of HDAC2 by the systemic administration of the HDAC inhibitor, trichostatin A (TSA), has neuroprotective potential against RGC damage [23]. To increase the potential of translating our results into clinical practice, we attempted to resolve issues regarding the side effects of systemically administered drugs and short retention time of intravitreally injected drugs. Thus, we prepared TSA-loaded liposomes based on a previous study [17]. The present study aimed to evaluate the neuroprotective effects of an intravitreal injection of TSA-loaded liposomes in a mouse model of optic nerve crush (ONC) and determine whether the TSA-loaded liposomes have therapeutic potential in RGC diseases.

## 2. Results

### 2.1. Residence Time of Intravitreally Injected TSA

**Figure 1:**
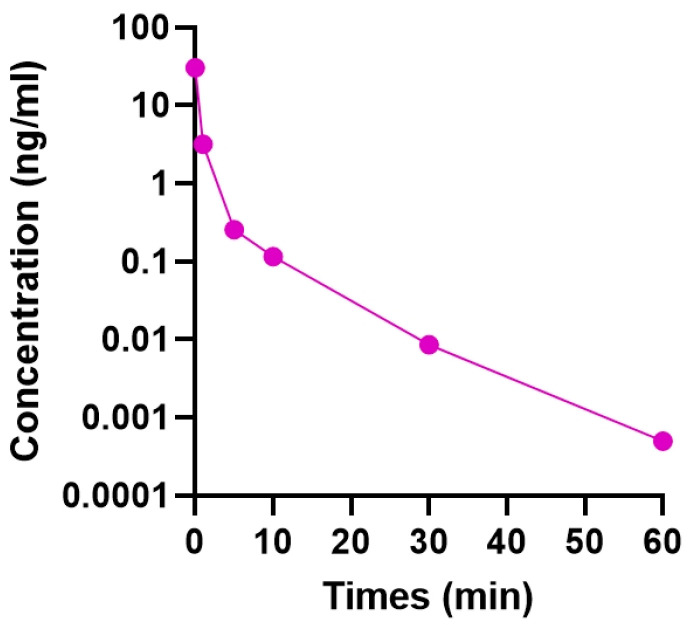
Concentration–time profile in the vitreous of mouse eye following the intravitreal injection of 1 μM trichostatin A (TSA).

We intravitreally injected 0.5 μL of 1 μM TSA (302.37 ng/mL) at a final concentration of 30.237 ng/mL, assuming the free intraocular volume of the mouse eye to be 5 μL [24]. Figure 1 shows the concentration of intravitreally injected TSA in the vitreous cavity at different time points. Consistent with a previous study [25], the injected TSA cleared from the eye rapidly and more than 99% of the dose was eliminated within 1 h after injection.

### 2.2. Liposomal Characterization

**Figure 2:**
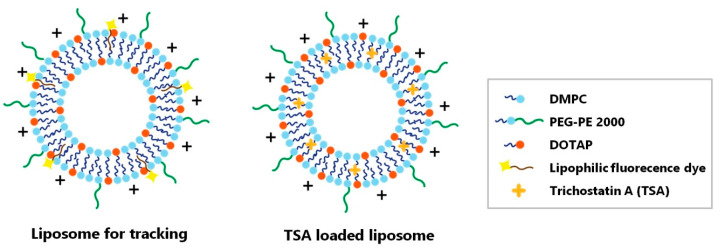
Schematic illustration of liposome for tracking and TSA-loaded liposome. Two liposome particles were synthesized. (Left) The liposome was synthesized for the purpose of tracking the location of the drug when it was administered to the vitreous cavity. (Right) The liposome was loaded with TSA and synthesized for the purpose of treatment. DMPC = 1,2-dimyristoyl-sn-glycero-3-phosphocholine; PEG 2000-PE = 1,2-distearoyl-sn-glycero-3-phosphoethanolamine-N-[methoxy(polyethylene glycol)-2000]; DOTAP = 1,2-dioleoyl-3-trimethylammonium propane.

**Figure 3:**
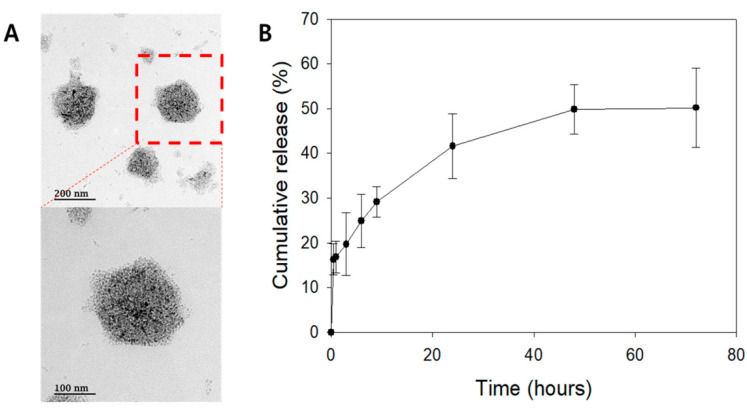
Liposome characteristics. (**A**) Transmission electron microscopy (TEM) image of nonloaded liposomes. (**B**) Cumulative TSA release profile of TSA-loaded liposomes.

Liposomes were produced as illustrated in Figure 2. The mean diameter of bare liposomes was 118.30 ± 45.06 nm, with a polydispersity index of 0.369 and zeta potential of 20.70 ± 6.25 mV. After loading TSA, the mean diameter of liposomes was 155.20 ± 57.37 nm, with a polydispersity index of 0.335 and zeta potential of 15.20 ± 5.95 mV. Transmission electron microscopy (TEM) images showed spherical liposomes that were predominantly unilamellar (Figure 3A). To maintain the liposome shape, TEM images were taken after loading commercialized dextran-coated iron oxide nanoparticles. The encapsulation efficacy (EE%) of liposomes, normalized to the lipid concentration, was 21.96% and corresponded to approximately 17.8 nM of TSA. The cumulative release profile of TSA for the TSA-loaded liposomes showed a 50% release for 72 h with initial 16% burst release (Figure 3B).

### 2.3. Temporal Distribution of Intravitreally Injected Liposomes

**Figure 4:**
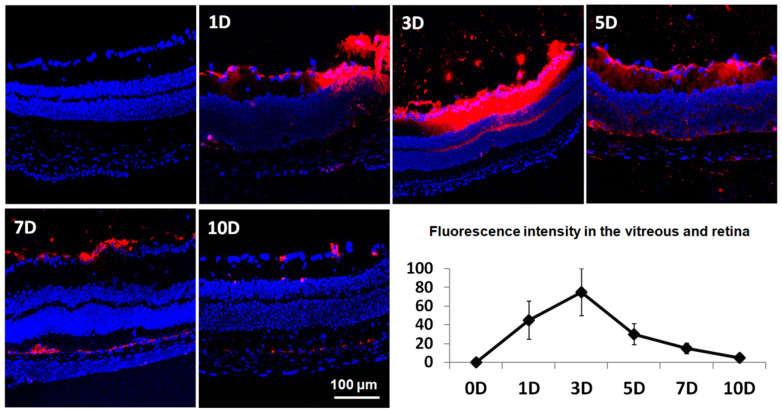
Temporal distribution of intravitreally injected liposomes. Images with lower magnification of the central retina including optic nerve head are presented in Supplementary Figure S1. Fluorescence microscopic images of the retina and fluorescence intensity at different time points after intravitreal injection of liposomes loaded with lipophilic fluorescence dye (red) are presented. The fluorescence intensity peaked at 3 days postinjection and declined gradually over a period of 10 days. D = days; Blue color = 4′,6-diamidino-2-phenylindole (DAPI) staining of retinal cell nuclei; Scale bar = 100 μm.

Figure 4 demonstrates the distribution of liposomes at 1, 3, 5, 7, and 10 days postinjection. The vitreous humor consists of hyaluronan, an anionic hydrophilic polymer with a high molecular weight, and collagen fibers that provide strength and resistance to tractional forces. As previously reported, polyethylene glycol (PEG)-ylated liposomes with moderate positive surface charge showed broad distribution throughout the vitreous humor, reaching the ganglion cell layer of the inner retina. The fluorescence intensity peaked at 3 days postinjection and declined gradually over a period of 10 days.

### 2.4. Intravitreal Injection of TSA-Loaded Liposomes Reduces Glial Cell Activation after ONC

**Figure 5:**
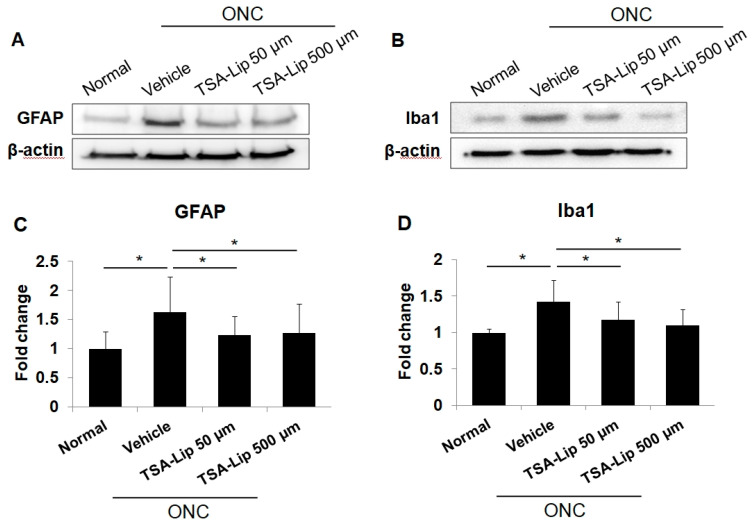
Effect of intravitreally injected TSA-loaded liposomes on gliosis in retina after optic nerve crush (ONC). The ONC procedure markedly increased GFAP (**A**,**C**) and Iba1 (**B**,**D**) protein expression in retinas at 3 days. The intravitreal injection of TSA-loaded liposomes significantly attenuated this response. The relative chemiluminescence intensity for each protein band was normalized using β-actin as a calibrator. TSA**-**Lip 50 µM = liposomes loaded with 50 µM TSA; TSA**-**Lip 500 µM = liposomes loaded with 500 µM TSA. Error bars represent the SD (*n* = 6 retinas per group); * *p* < 0.05; one-way ANOVA with post-hoc LSD test.

**Figure 6:**
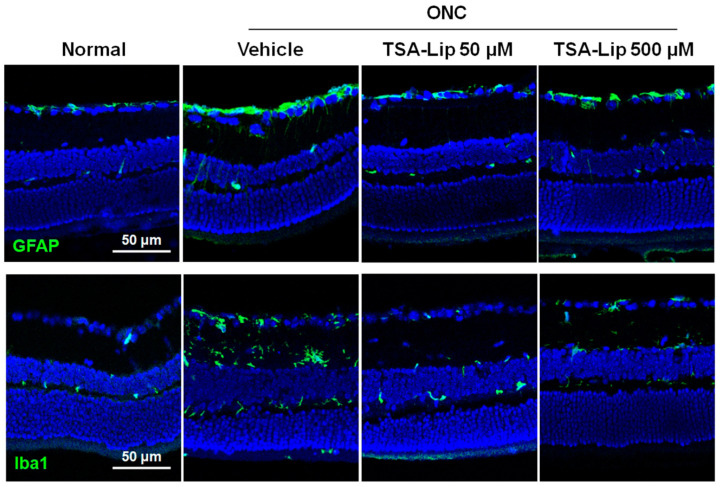
Immunohistochemical staining of GFAP and Iba1 in retinas at 7 days after ONC. (Upper) Sections from vehicle-injected retinas after ONC show increased GFAP activity on the RGC layer and the radial process of macroglial cells spanning the inner plexiform layer. The intravitreal injection of TSA-loaded liposomes significantly attenuated the GFAP activity in retinas after ONC. (Lower) Immunolabeling for Iba1 in retinal sections showed prominent activation of microglia in vehicle-injected retinas after ONC. This activation was ameliorated in the retinas injected with TSA-loaded liposomes. Images with lower magnification are presented in Supplementary Figure S2. TSA**-**Lip 50 µM = liposomes loaded with 50 µM TSA; TSA**-**Lip 500 µM = liposomes loaded with 500 µM TSA.

Glial fibrillary acidic protein (GFAP) is a marker of macroglial cells such as Müller cells and astrocytes. Ionized calcium binding adaptor molecule-1 (Iba1) is a marker of microglial cells. It has been demonstrated that reactive glial activation in the retina is linked to the expression of various proinflammatory mediators, and thus, it results in RGC death [26,27]. In this study, western blot analysis showed that the expression of GFAP and Iba1 was markedly increased at 3 days after ONC injury, and the intravitreal injection of TSA-loaded liposomes significantly attenuated the ONC-induced increase in GFAP and Iba1 (*n* = 6 retinas per group, Figure 5). Figure 6 shows the results of immunohistochemical staining for GFAP and Iba1, following ONC induction. There were no significant differences in glial expression between the two different doses of TSA-loaded liposome groups.

### 2.5. Intravitreal Injection of TSA-Loaded Liposomes Suppresses RGC Apoptosis after ONC

**Figure 7:**
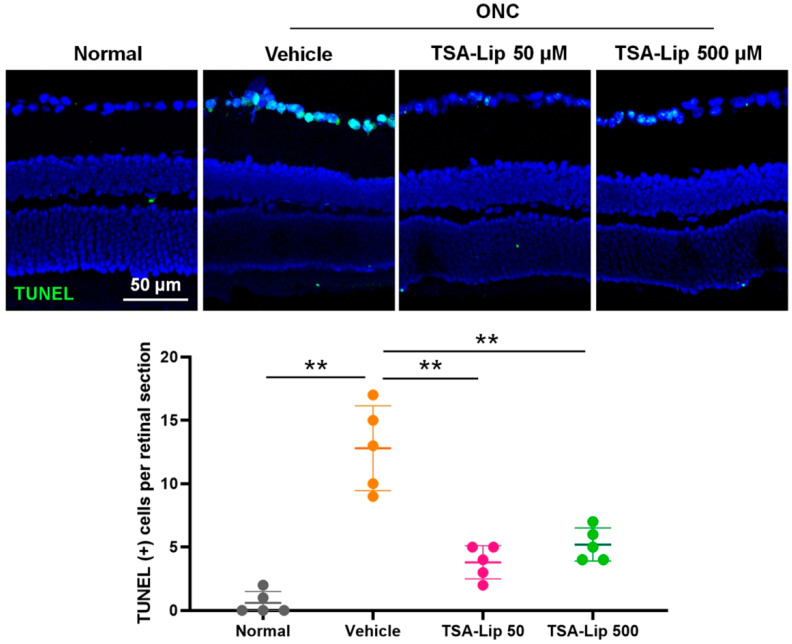
TUNEL staining was performed on retinal sections 7 days after ONC. Vehicle-injected retinas demonstrated significantly higher TUNEL-positive apoptotic cells in RGC layer after ONC (** *p* < 0.001, compared to the uninjured normal eyes). The intravitreal injection of TSA-loaded liposomes immediately after ONC injury, suppressed RGC apoptosis (** *p* < 0.001, compared to the vehicle group). There were no significant differences in TUNEL-positive apoptotic cells between the groups with 50 μM and 500 μM TSA-loaded liposomes. Images with lower magnification are presented in Supplementary Figure S3. TSA**-**Lip 50 µM = liposomes loaded with 50 µM TSA; TSA**-**Lip 500 µM = liposomes loaded with 500 µM TSA. All comparisons were performed using one-way ANOVA with post-hoc LSD test.

Apoptosis promotes retinal cell depletion after ONC injury. RGC apoptosis was measured by TUNEL assay in retinal sections on day 7 after ONC induction. TUNEL staining revealed significant differences in reactivity in the ganglion cell layer between the vehicle injection group and the TSA-loaded liposomes injection group (*n* = 5 retinas per group, Figure 7). There were no statistically significant differences in TUNEL-positive cells between the groups with 50 μM and 500 μM TSA-loaded liposomes.

### 2.6. Effect of Intravitreally Injected TSA-Loaded Liposomes on RGC Survival after ONC

**Figure 8:**
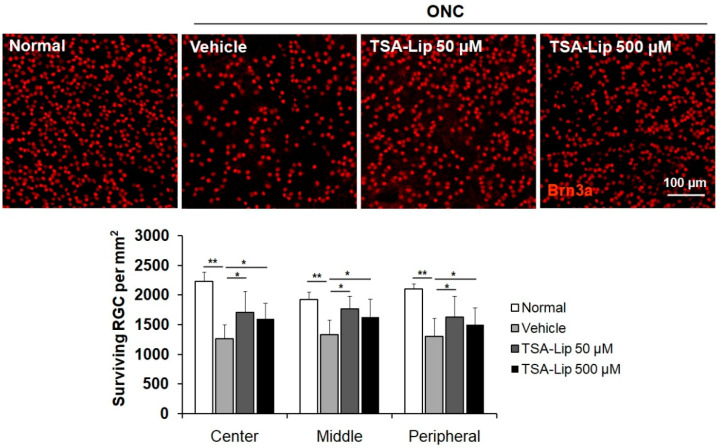
Effect of TSA-loaded liposomes on RGC survival after ONC. Retinal flat mounts are shown for the uninjured normal retinas, vehicle-injected retinas, and retinas injected with 50 μM and 500 μM TSA-loaded liposomes (*n* = 5 retinas per group). A quantitative analysis of RGC survival in center, middle, and peripheral zones of each retinal quadrant is also shown as a graph. TSA**-**Lip 50 µM = liposomes loaded with 50 µM TSA; TSA**-**Lip 500 µM = liposomes loaded with 500 µM TSA. Scale bar = 100 µm; ** *p* < 0.001, compared to the uninjured normal retina; * *p* < 0.05 compared to the vehicle-injected retina; one-way ANOVA with post-hoc LSD test.

We evaluated RGC survival by immunohistochemistry of retinal whole mounts with anti-Brn3a antibody labeling. As shown in Figure 8, whole retinas showed significant RGC loss at 14 days after ONC. Intravitreal injection of TSA-loaded liposomes prevented RGC loss in the center, middle, and peripheral retinal area relative to vehicle-treated retinas. No significant differences in Brn3a-positive cells were observed between the different dosage groups of TSA-loaded liposomes (*n* = 5 retinas per group).

## 3. Discussion

Acetylation and deacetylation of histone is an important mechanism to regulate gene expression and chromatin remodeling. A balanced histone acetyltransferase (HAT) and histone deacetylase (HDAC) interplay is vital for neuronal cell functionality, thereby coordinating gene expression in a healthy manner. During a neurodegenerative state, this balance is altered, and HDAC activity overrides HAT activity [28,29]. HDACs and their inhibitors have emerged as promising targets for regulating neurodegenerative disorders. Pharmacological intervention with HDAC inhibitors has been shown to be neuroprotective in various neurodegenerative models, both in vivo and in vitro [23,30,31,32]. In a recent study, our group also showed that an intraperitoneal injection of TSA effectively facilitated recovery from ischemia-reperfusion injury-induced RGC death [22].

An important challenge in the application of neuroprotective agents in RGC diseases is the development of a safe and effective means of long-lasting delivery of drugs to the target cells. We considered that an intravitreal injection of TSA might enhance its efficacy by increasing its concentration in the vitreous cavity, while reducing the risk of side effects associated with systemic dosing regimens. However, because the drug remains in the eye for only a short duration, it needs to be injected frequently into the vitreous. It has been reported that the half-life of intravitreally injected TSA in the rabbit eye was in the range of 1.7 to 3.3 h [25]. In the present study, we found that the intravitreal half-life of TSA was shorter, and more than 99% of the dose was eliminated within 1 h after injection. To overcome the short retention time of intravitreally injected TSA, the drug was incorporated into a liposome, and novel TSA-loaded liposomes were developed. Liposomes were chosen because they have almost no toxicity, have low antigenicity, can be biodegradable and metabolizable in vivo, can be easily modified to facilitate targeting, and can hold various types of drugs with different properties, such as lipophilic or hydrophilic molecules [17,18,19,20,21,22].

When developing nanoparticles for intravitreal injection, the first requirement is that the nanoparticles should have optimal mobility inside the vitreous cavity. The vitreous consists mainly of water, negatively charged hyaluronic acid, hydrophobic collagen fibrils, and a few peripheral hyalocytes, and can present a significant barrier to intravitreally administered drugs [33]. It has been reported that the interference of the vitreal network in the mobility of the particles is dependent on the size and surface characteristics of the particles [34,35]. The surface charge of liposome formulations can be adjusted by modifying the molar ratio of the cationic phospholipid 1,2-dioleoyl-3-trimethylammonium propane (DOTAP), and the effect of particle size and surface characteristics on interactions with the vitreous humor has been investigated previously [17]. Lee et al. [17] demonstrated that PEGylated liposomes with moderate positive surface charge showed a uniform distribution in the vitreous humor and effectively reached the inner retinal layers without premature clearance. In this study, we also found that intravitreally administered PEGylated liposomes with a diameter of 118 nm and moderately positive surface charge effectively reached the inner retina. Importantly, the fluorescence of tracking liposomes was detected up to 10 days after intravitreal injection, with peak intensity at 3 days postinjection.

Injured RGCs and their glial environment are equipped with counteractive measures in various neurodegenerative diseases. Although Müller cells, astrocytes, and microglial cells have different developmental origins, they share many functions within the retina [27]. Under pathological conditions, they are activated, undergo functional and morphological changes associated with gliosis, and potentially exacerbate the death of RGCs. Therefore, reversal of reactive gliosis has been demonstrated to be neuroprotective. Recently, our group reported that this glial activation is closely associated with HDAC2 activity in retinas, and that HDAC inhibition by TSA has a neuroprotective effect on RGC by decreasing reactive gliosis [23]. Similarly, in the current study, we observed increased macroglial (GFAP) and microglial (Iba1) expression and RGC death in the retinas after ONC. The intravitreal injection of TSA-loaded liposomes significantly decreased ONC-induced reactive gliosis and increased RGC survival.

There is growing evidence that HDACs play an important role in neuroinflammation. It has been reported that HDACs regulate the inflammatory response of mixed glial cultures and macrophages [36,37]. In addition, various HDAC inhibitors have known to be associated with increased gene transcription encoding neurotrophic factors and decreased gene transcription inducing neuronal apoptosis [37]. However, the mechanism by which intravitreally injected TSA leads to reduced gliosis and increased RGC survival after ONC will require further examination, as specific intracellular signaling pathways in RGCs are yet to be determined.

Apoptosis, demonstrated by DNA fragmentation, has been described as a mechanism of RGC death after ONC [38]. TUNEL staining showed that TSA treatment successfully decreased the apoptosis of RGCs after ONC. Our results suggest that the neuroprotective effect of intravitreally administered TSA is partly mediated by suppressing ONC-induced RGC apoptosis.

Pelzel et al. [39] previously reported that 1 μL of intravitreally injected 20 μM TSA immediately after ONC exerted a modest protective effect on RGCs. We determined the concentration of TSA based on their study. In the present study, to minimize the transient elevation of intraocular pressure after intravitreal injection, we injected a smaller volume of solution (0.5 μL of TSA-loaded liposomes) with a TSA concentration of 50 μM (not 40 μM as in the previous study). The concentration was higher than that used in the previous study, because the incorporated TSA would not be released from the liposomes simultaneously. The intravitreal administration of 0.5 μL of TSA-loaded liposomes immediately after ONC was found to protect RGC from injury. Interestingly, a 10-fold higher dose of 500 μM TSA-loaded liposomes did not show superior neuroprotective effect on RGCs, i.e., it did not follow linear pharmacokinetics. The exact mechanism for nonlinear pharmacokinetics should be studied further. Our results may be associated with additional cytotoxic effects of higher concentrations of TSA. While beyond the scope of this preliminary evaluation, it is essential to acquire pharmacokinetic and safety data for TSA-loaded liposomes in future studies, to rule out significant drug toxicity before clinical application.

Our study has several limitations. While liposomes are known to be nontoxic in vivo, a more definitive experiment to completely rule out the drug toxicity might be needed. In addition, we did not evaluate the mechanisms of RGC survival and decreased gliosis after intravitreal injection of TSA. The exact molecular mechanism remains to be clarified by further experiments. Long-term evaluation of neuroprotective effects after intravitreal injection of TSA-loaded liposomes also remains to be investigated.

## 4. Materials and Methods

### 4.1. Animal Use

All experiments were conducted in accordance with the “Statement for the Use of Animals in Ophthalmic and Vision Research” described by “The Association for Research in Vision and Ophthalmology” (ARVO). The protocol was approved by the “Institutional Animal Care and Use Committee” of Chonnam National University Hospital (CNU IACUC –H-2018-19; approval date = 27 April 2018). C57BL/6J mice, obtained at 3 months of age (20–25 g in weight), were housed in individual cages under controlled lighting conditions (12 h light/12 h dark) and provided tap water and food ad libitum throughout the study. In this experiment, we used C57BL/6J mice, because they have been widely used and studied as a model for optic nerve crush injury [40,41,42,43]. All surgical procedures were performed under anesthesia, and every effort was made to minimize animal suffering.

### 4.2. Determination of Intravitreal Residence Time of TSA

Ocular pharmacokinetic studies in mice are challenging, because the volume of vitreous in an adult mouse is only 5 μL. Therefore, we attempted to determine the residence time of intravitreally injected TSA in mice, as there are no data regarding the same. C57BL/6J mice were anesthetized with a mixture of 40 mg/kg of ketamine hydrochloride (Yuhan, Seoul, Korea) and 4 mg/kg of xylazine hydrochloride (Rompun^®^; Bayer Korea, Seoul, Korea) by intramuscular injection. Next, 0.5 μL of 1 μM TSA was intravitreally injected into the mice. The mice were sacrificed via cervical dislocation at 1, 5, 10, 30, and 60 min postinjection and the vitreous from each eye was eviscerated as described previously [44]. Briefly, scleral tissue posterior to the limbus was grasped with forceps and a microsurgical blade was used to make a linear incision in the cornea from limbus to limbus. A fine curved needle holder was inserted behind the lens and then pulled forward, eviscerating both the lens and vitreous. The vitreous tissue was centrifuged with 20 μL of protease inhibitor cocktail (Roche Applied Science; Indianapolis, IN, USA) dissolved in phosphate buffered saline (PBS).

The concentration of TSA in the vitreous was measured through liquid chromatography-tandem mass spectrometry (API 4000Q TRAP; AB SCIEX, Foster City, CA, USA). The mass spectrometer was equipped with an electrospray ionization (ESI) source. It was set up in positive and multiple reaction monitoring (MRM) modes, monitoring the transitions of 303.2/148.2. The ion spray voltage was set to 5500 V and the probe temperature was set at 500 °C. The curtain gas, nebulizer (GS1), and turbo gas (GS2) were set to 30, 60, and 60 psi, respectively. Liquid chromatography was performed on the Shimadzu LC 20A system. The mobile phase was 60% acetonitrile, 40% water, and 0.1% formic acid at a flow rate of 0.25 mL/min. The column was Phenomenex Gemini C18 with a pore size of 3.0 μm, and internal diameter of 50 mm × 2.0 mm, with Gemini C18 (4.0 mm × 2.0 mm) guard cartridge. The column oven was set to 40 °C.

### 4.3. Study Design

The study included two sets of experiments. First, we tested the distribution and residence time of liposomes after intravitreal injection. Liposomes loaded with the lipophilic fluorescent dye were injected and the mice retinas were harvested at 1, 3, 5, 7, and 10 days postinjection (*n* = 4 mice per time point). The confocal fluorescence microscopic images were obtained at the same parameter settings from the cryostat sections of enucleated eyes. Each eye was sectioned to be 10-12μm thick, and 5 slides per eye were chosen to determine the distribution of liposomes and calculate the average fluorescence intensity in the vitreous and retina. The fluorescence was determined with ImageJ (version 1.49, http://imagej.nih.gov/ij/; provided in the public domain by the National Institutes of Health, Bethesda, MD, USA).

Second, the mice were divided into four groups for analysis: (1) normal eyes with uninjured optic nerve (normal control, *n* = 16); (2) eyes that underwent the ONC procedure and received an intravitreal injection of vehicle solution (vehicle control, *n* = 16); (3) eyes that underwent the ONC procedure and received an intravitreal injection of liposomes loaded with 50 μM TSA (TSA**-**Lip 50 µM, *n* = 16); (4) eyes that underwent the ONC procedure and received an intravitreal injection of liposomes loaded with 500 μM TSA (TSA**-**Lip 500 µM, *n* = 16). The volume injected into the eyes was always the same and only the concentration of the TSA changed. The retinas were harvested for western blot analysis at 3 days after ONC, for immunohistochemical analysis and terminal deoxynucleotidyl transferase dUTP nick-end labeling (TUNEL) assay at 7 days after ONC, and for Brn3a staining at 14 days after ONC. The experiment was repeated in two sets to check for reproducibility.

### 4.4. Liposome Preparation and Trichostatin A Loading

1,2-dimyristoyl-sn-glycero-3-phosphocholine (DMPC); 1,2-distearoyl-sn-glycero-3-phosphoethanolamine-N-[methoxy(polyethylene glycol)-2000] (DSPE-PEG2000); and 1,2-dioleoyl-3-trimethylammonium propane (DOTAP) were obtained from Avanti Polar Lipids, Inc. (Alabaster, AL, USA). Lipophilic fluorescence dye CytoFlamma^®^ 552 (BioActs, South Korea) was incorporated for the purpose of tracking the location of liposomes. The lipid film was prepared with the lipid mixture at a molar ratio of 3.8% DSPE-PEG200: 76.2% DPPC: 20% DOTAP and at a molar ratio of 80% DMPC: 20% DOTAP. The film was then hydrated with PBS. The particle characteristics used in this study were based on the previous work by Lee et al. [17].

Trichostatin A (TSA)-loaded liposomes were synthesized. Briefly, a thin lipid film containing TSA was prepared using a lipid mixture at a molar ratio of 3.8% DSPE-PEG2000: 76.2% DPPC: 20% DOTAP with 25 µg of TSA per mL of liposome solution. The film was then hydrated with PBS. The concentration of TSA was determined by dissolving the liposomes in methanol and analyzing it using UV spectroscopy.

### 4.5. Optic Nerve Crush Model

C57BL/6J mice were anesthetized with a mixture of 40 mg/kg of ketamine hydrochloride (Yuhan, Seoul, Korea) and 4 mg/kg of xylazine hydrochloride (Rompun^®^; Bayer Korea, Seoul, Korea) by intramuscular injection. A drop of local anesthesia, proparacaine hydrochloride (Alcaine^®^; Alcon Laboratories, Inc., Fort Worth, TX, USA), was also given before the surgery. The intra-orbital optic nerve crush (ONC) was performed as described previously [45,46,47]. Briefly, the superior bulbar conjunctiva of the mice was incised and the optic nerve of the left eye was carefully exposed, while avoiding bleeding. Next, the optic nerve was crushed with cross-action forceps 1 to 2 mm posterior to the lamina cribrosa for 10 s, taking care to separate the dura mater and underlying retinal artery before crushing. After surgery, the fundi were checked to ensure that the retinal blood flow was intact. The left optic nerves were crushed in all the mice while right eyes were kept intact. An antibiotic ointment was applied to both eyes to prevent drying of cornea and infection.

### 4.6. Intravitreal Injection

After general anesthesia, the eyes were dilated with one drop of a combination of 0.5% tropicamide and 0.5% phenylephrine (Mydrin-P^®^; Santen, Osaka, Japan). Next, 0.5 μL of TSA-loaded liposomes was injected into the vitreous cavity using a Hamilton syringe (Hamilton Company, Reno, NV, USA) immediately after the ONC procedure. The control group mice received intravitreal injections of 0.5 μL of vehicle solution at the same time points.

### 4.7. Western Blot Analysis

For western blot analysis, whole retinas were used immediately or frozen at −70 °C until use. Retinal tissues were homogenized in a glass-Teflon Potter homogenizer in PRO-PREP^TM^ lysis buffer (iNtRoN Biotechnology, Inc., Seoul, Korea). Next, 10 µg of each sample was separated on a 10% polyacrylamide mini gel. After protein transfer, the membranes were blocked for 1 h at room temperature in Tris-buffered saline–Tween-20 solution [TBS-T; 10 mM Tris-HCl (pH 7.6), 150 mM NaCl, and 0.1% Tween-20] containing 5% non-fat dry milk. After blocking, the membranes were incubated overnight at 4 °C with a primary antibody against GFAP (1:3000; Cell Signaling Technology, Inc., Danvers, MA, USA), Iba1 (1:1000; Santa Cruz Biotechnology, Inc., Santa Cruz, CA, USA), and β-actin (1:10,000; Sigma, MO, USA). After three washes with TBS-T, the membranes were incubated for 1 h at room temperature with a peroxidase-conjugated goat anti-mouse IgG (1:3000; Santa Cruz Biotechnology, Inc., Santa Cruz, CA, USA) or peroxidase-conjugated goat anti-rabbit IgG (1:3000; Cell Signaling Technology, Inc., Danvers, MA, USA) in TBS-T containing 5% non-fat dry milk. The signals were visualized by enhanced chemiluminescence and quantified using an LAS-3000 image analyzer (Fujifilm, Tokyo, Japan).

### 4.8. Immunohistochemical Analysis

Immunohistochemical staining of 7 µm sections of full-thickness retinas was performed by immunofluorescence with the following primary antibodies: mouse monoclonal anti-GFAP antibody (1:200; Cell Signaling Technology, Inc., Danvers, MA, USA) and mouse monoclonal anti-Iba1 antibody (1:150; Santa Cruz Biotechnology, Inc., Santa Cruz, CA, USA). Seven days after ONC, the eyes were enucleated and fixed in freshly made 4% paraformaldehyde in 0.1 M PBS for 1 h, and then the free retina was harvested and flattened. The tissues were dehydrated and embedded in paraffin, and the retinal cross sections were then obtained. The tissues were blocked with 1% bovine serum albumin in PBS for 1 h at room temperature to prevent nonspecific background staining, and then with the primary antibodies overnight at 4 °C. The sections were washed several times with PBS, incubated with Alexa Fluor^®^ 488-conjugated chicken anti-mouse IgG (1:250; Invitrogen, Carlsbad, CA, USA) or Alexa Fluor^®^ 546-conjugated rabbit anti-goat IgG (1:250; Invitrogen, Carlsbad, CA, USA) for 4 h at 4 °C, and then washed again with PBS. The sections were counterstained with 0.1 µg/mL of Hoechst 33,342 stain in PBS (Invitrogen, Carlsbad, CA, USA). The images were analyzed using a Zeiss LSM 800 confocal microscope (Carl Zeiss, Jena, Germany).

### 4.9. TUNEL Assay

Apoptotic cells were studied in retinal samples collected 7 days after ONC using a TdT-mediated dUTP nick-end labeling (TUNEL) assay kit (In Situ Cell Death Detection with Fluorescein; Roche Biochemicals, Mannheim, Germany), according to the manufacturer’s protocol. The cross-sections from central retina were used. All sections for TUNEL assay were chosen from the same area of the retina. Two cross sections per eye were used to collect the images and TUNEL-positive cells were quantified manually on each retinal section. The tissue sections were examined with a Zeiss LSM 800 confocal microscope.

### 4.10. Retinal Wholemounts and Brn3a Staining

Two weeks after ONC, the retinas were dissected from enucleated eyes and flattened. The retinas were immersed in PBS containing 30% sucrose for 24 h at 4 °C, frozen for 15 min at −70 °C, blocked in PBS containing 1% bovine serum albumin and 0.5% Triton X-100 and incubated with a polyclonal goat anti-Brn3a antibody (1:100; Santa Cruz Biotechnology, Inc., Santa Cruz, CA, USA) for 72 h at 4 °C. After several washes, the retinas were incubated with the secondary antibody, Alexa Fluor^®^-568-conjugated donkey anti-goat IgG antibody (1:250; Invitrogen, Carlsbad, CA, USA,) for 4 h, and subsequently washed with PBS. To evaluate the loss of RGCs, each retinal quadrant was divided into three zones—center, middle, and peripheral retina—corresponding to 1/6, 3/6, and 5/6 of the retinal radius, respectively. RGCs were counted in 32 distinct areas of 0.25 mm^2^ (two areas in the center, three areas in the middle, and three areas at the periphery of each retinal quadrant) by two investigators in a blinded fashion, and the scores were averaged. To ensure that similar regions were assessed in each eye and avoid any possible area prejudice, a reference nasal mark was made on the retina when harvesting and all retinas were oriented in the same way. The fluorescent images of the tissues were acquired and analyzed using confocal microscopy on a laser scanning microscope.

### 4.11. Statistical Analysis

The data are presented as the mean ± standard deviation (SD). One-way analysis of variance (ANOVA) with the appropriate least significant difference (LSD) post-hoc test was used to compare the experimental groups. The analyses were performed using the SPSS software version 21.0 for Windows (SPSS Inc., Chicago, IL, USA). Values of *p* < 0.05 were considered statistically significant.

## 5. Conclusions

In this study, we demonstrated that the incorporation of TSA in PEGylated liposomes with surface positive charge prolonged TSA residence time in the retina to about 10 days. An intravitreal injection of TSA-loaded liposomes reduced retinal gliosis and RGC apoptosis in a mouse model of ONC. The neuroprotective effect of TSA-loaded liposomes may be translatable to clinical applications, although further studies are warranted.

## Supplementary Materials

The following are available online at https://www.mdpi.com/1422-0067/21/23/9297/s1.
