# Supplementary material for: Intravitreal Injection of Liposomes Loaded with a Histone Deacetylase Inhibitor Promotes Retinal Ganglion Cell Survival in a Mouse Model of Optic Nerve Crush
Source: Int J Mol Sci. 2020 Dec 6;21(23):9297. doi: 10.3390/ijms21239297 (PMC7730870; doi:10.3390/ijms21239297)
Supplement: Supplementary file 1 [file ijms-21-09297-s001.pdf]

Intravitreal injection of liposomes loaded with a histone deacetylase inhibitor promotes retinal ganglion cell survival in a mouse model of optic nerve crush

Mi Sun Sung,[1] Myeong Ju Moon,[2] Reju George Thomas,[2] So Young Kim,[1] Junsung Lee,[3] Yong Yeon Jeong,[2] In-Kyu Park,[4] and Sang Woo Park[1]

[1] Department of Ophthalmology, Chonnam National University Medical School and Hospital, Gwangju, South Korea

[2] Department of Radiology, Biomolecular Theranostics (BiT) Lab, Chonnam National University Medical School, Hwasun, South Korea

[3] Parangsae Eye Clinic, Gwangju, South Korea

[4] Department of Biomedical Sciences, Chonnam National University Medical School, Hwasun, South Korea

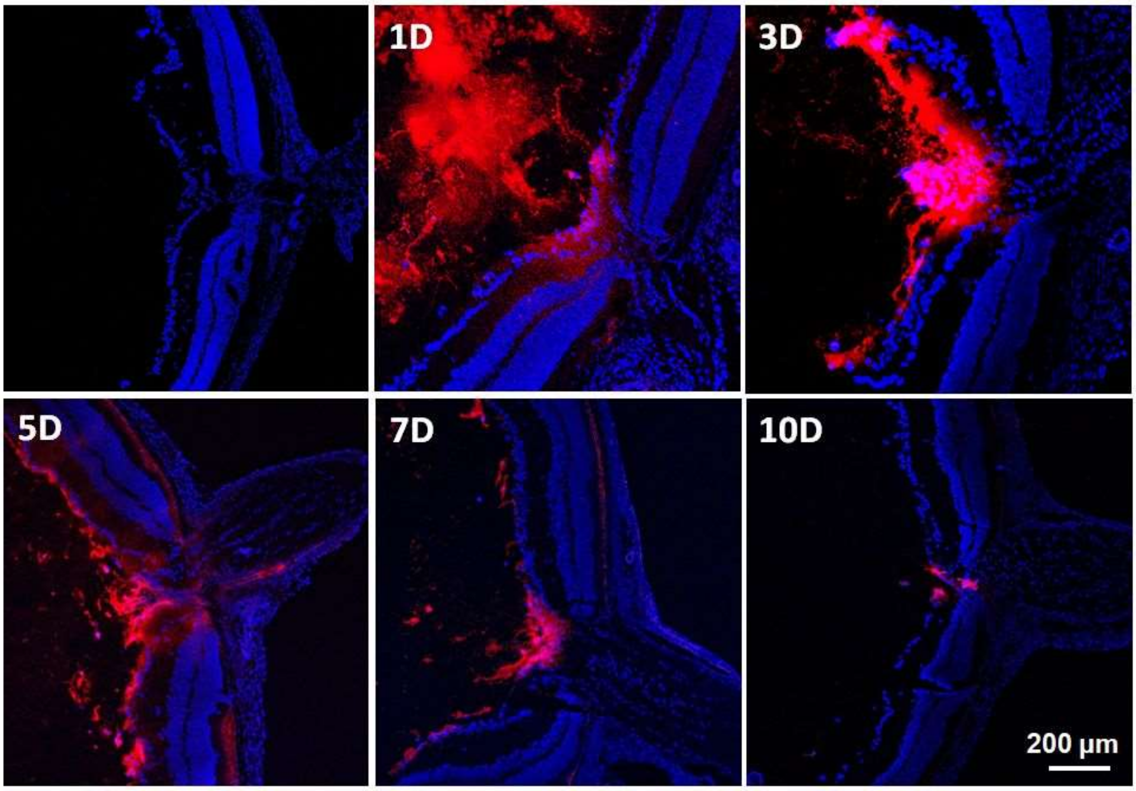

Supplementary Figure S1. Temporal distribution of intravitreally injected liposomes with lower magnification. Images were taken from the central retina including optic nerve head. Fluorescence microscopic images of the retina at different time points after intravitreal injection of liposomes loaded with lipophilic fluorescence dye (red) are presented. Blue color = 4′,6-diamidino-2-phenylindole (DAPI) staining of retinal cell nuclei; Scale bar = 200 µm

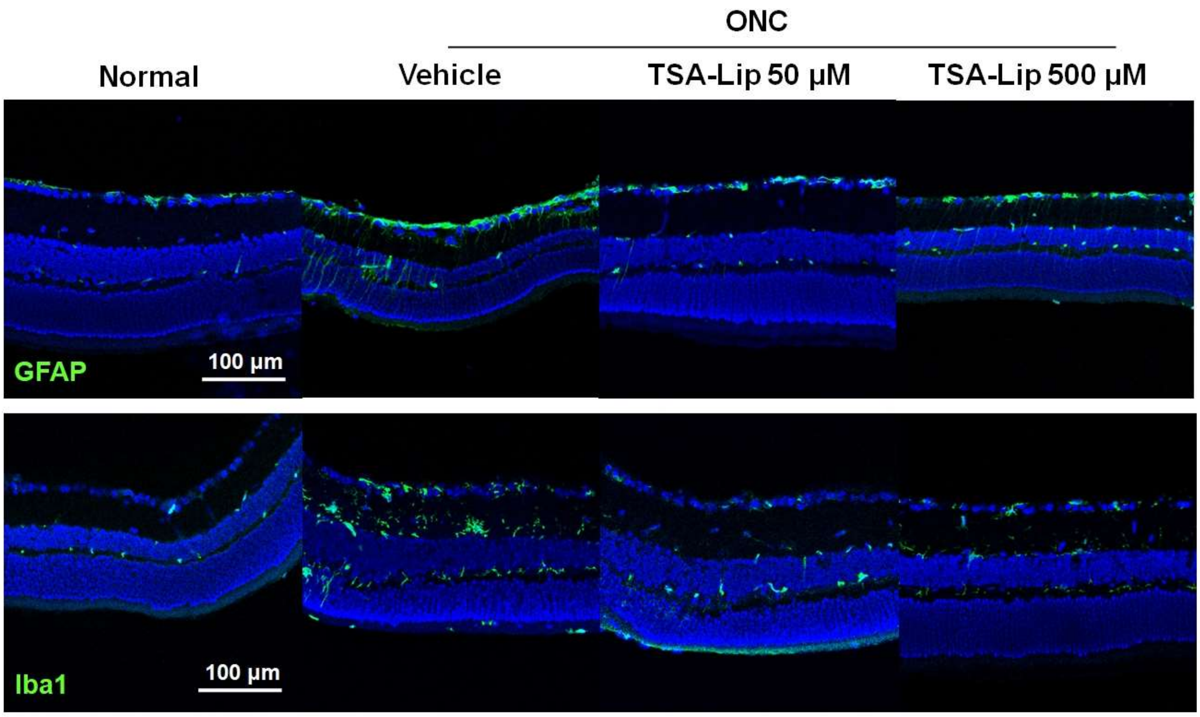

Supplementary Figure S2. Immunohistochemical staining images with lower magnification for GFAP and Iba1 in retinas at 7 days after ONC. TSA-Lip 50 μM = liposomes loaded with 50 μM TSA; TSA-Lip 500 μM = liposomes loaded with 500 μM TSA; Scale bar = 100 μm.

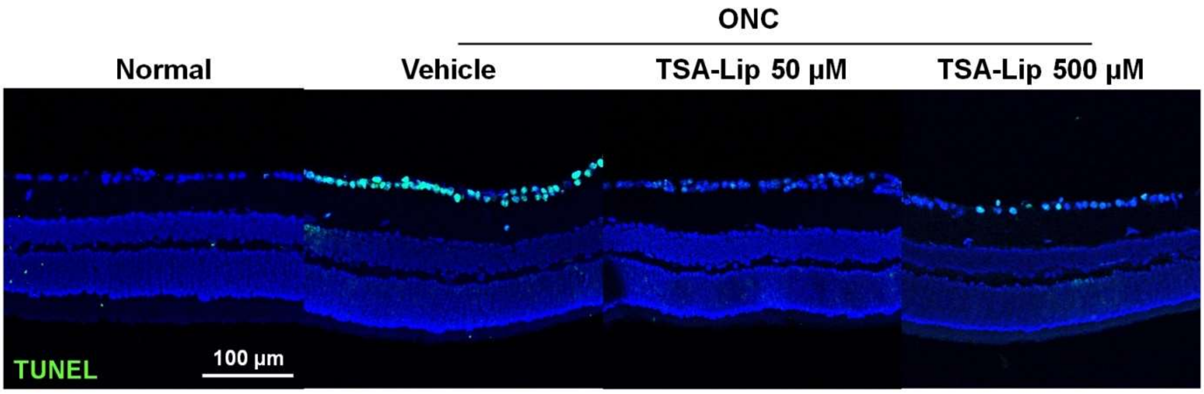

Supplementary Figure S3. TUNEL staining images with lower magnification at 7 days after ONC. TSA-Lip 50 μM = liposomes loaded with 50 μM TSA; TSA-Lip 500 μM = liposomes loaded with 500 μM TSA.
